# Post‐Dispersal Embryo Growth Is a Thermal Checkpoint for Seed Regeneration

**DOI:** 10.1002/ece3.73978

**Published:** 2026-07-07

**Authors:** Keyvan Maleki, Elias Soltani

**Affiliations:** ^1^ Department of Horticulture and Crop Science The Ohio State University Columbus Ohio USA; ^2^ Department of Agronomy and Plant Breeding Sciences, College of Aburaihan University of Tehran Tehran Iran

**Keywords:** embryo‐growth thermal niche, germination ecology, phenological mismatch, phylogenetic conservatism, thermal performance curves

## Abstract

Many seeds with underdeveloped embryos, particularly those exhibiting morphological (MD) or morphophysiological dormancy (MPD), must complete a phase of post‐dispersal embryo growth before germination can occur. Yet this developmental stage is typically treated as a passive delay within dormancy frameworks rather than as an independent ecological process. We propose that embryo growth constitutes a distinct thermal performance process governed by species‐specific base, optimum, and ceiling temperatures that are mechanistically decoupled from germination thresholds. As such, embryo growth functions as a developmental checkpoint that filters regeneration under variable climatic conditions. We introduce the concept of the embryo‐growth thermal niche (EGTN), defined by (i) a temperature‐dependent initiation threshold and (ii) a thermal performance curve governing elongation rates once growth has begun. The EGTN determines not only the pace of embryo development but the climatic window within which regeneration is possible. This framework generates counterintuitive predictions under climate warming. Because embryo growth may decline or arrest at supra‐optimal temperatures, warming can reduce cumulative developmental progress and delay seedling emergence, even when germination rates increase, producing phenological inversion relative to linear thermal‐time expectations. Viewing embryo growth as a functional trait reveals distinct regeneration strategies structured by thermal breadth and upper thermal limits, independent of dormancy class. We further predict strong phylogenetic signal in embryo‐growth thermal parameters, potentially constraining adaptive responses to rapid climate change. By integrating developmental biology with functional trait ecology, the EGTN framework positions post‐dispersal embryo growth as a central determinant of regeneration timing, climate sensitivity, and evolutionary constraint. Incorporating embryo‐growth thermal niches into regeneration models will improve forecasts of plant responses in a warming world.

## Graphical abstract

Seeds with underdeveloped embryos must complete a phase of post‐dispersal embryo growth before radicle emergence, yet this developmental stage is typically treated as a passive delay within dormancy frameworks rather than as an independent ecological process.

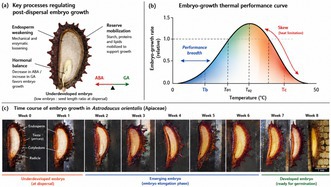

“In seeds with underdeveloped embryos, germination is impossible until the embryo has completed a definite phase of growth, the rate and possibility of which are strictly limited by temperature conditions”. (Nikolaeva 1977).

## 1 Introduction

Seeds with underdeveloped embryos are characterized by a low embryo‐to‐seed length ratio at dispersal (Baskin and Baskin 2014). In many species exhibiting morphological (MD) or morphophysiological dormancy (MPD), these embryos must complete a phase of post‐dispersal growth before germination can occur. This requirement characterizes a wide range of angiosperms (Nikolaeva 1977; Rosbakh et al. 2023), although the mechanisms regulating embryo growth differ among dormancy classes. In MD seeds, embryo growth proceeds once seeds are exposed to suitable environmental conditions, whereas in MPD seeds an additional physiological dormancy component must be alleviated before, during, or in conjunction with embryo growth (Baskin and Baskin 2014). Consequently, dormancy release and embryo development may be governed by distinct thermal requirements or may occur simultaneously, depending on species and dormancy level (Nikolaeva 1977; Baskin and Baskin 2014). Despite this diversity, embryo growth remains a necessary developmental stage linking dormancy regulation to germination, yet it remains weakly integrated into theories of germination strategy and regeneration timing (Walker et al. 2026). Current regeneration models implicitly assume that embryo development mirrors germination thermal responses. We argue that this assumption is mechanistically incomplete.

In most frameworks, embryo growth is treated as a passive developmental delay or absorbed into dormancy‐release processes, despite growing evidence that these processes are thermally and mechanistically decoupled (Blandino et al. 2025). We propose that post‐dispersal embryo growth constitutes a distinct thermal checkpoint within the broader dormancy–germination continuum. Although embryo growth has long been recognized as an important component of MD and MPD (Bewley et al. 2013; Baskin and Baskin 2014), its temperature‐dependent dynamics are rarely considered explicitly. Rather than viewing embryo growth solely as a developmental phase preceding germination, we argue that it can be conceptualized as a thermal performance process that influences the timing and likelihood of regeneration under different climatic conditions. Recognizing embryo growth as a thermal checkpoint reframes it from a preparatory phase to a strategic developmental filter with direct consequences for phenology, climate sensitivity, and evolutionary constraint.

## 2 Embryo Growth as a Thermal Performance Process

**FIGURE 1:**
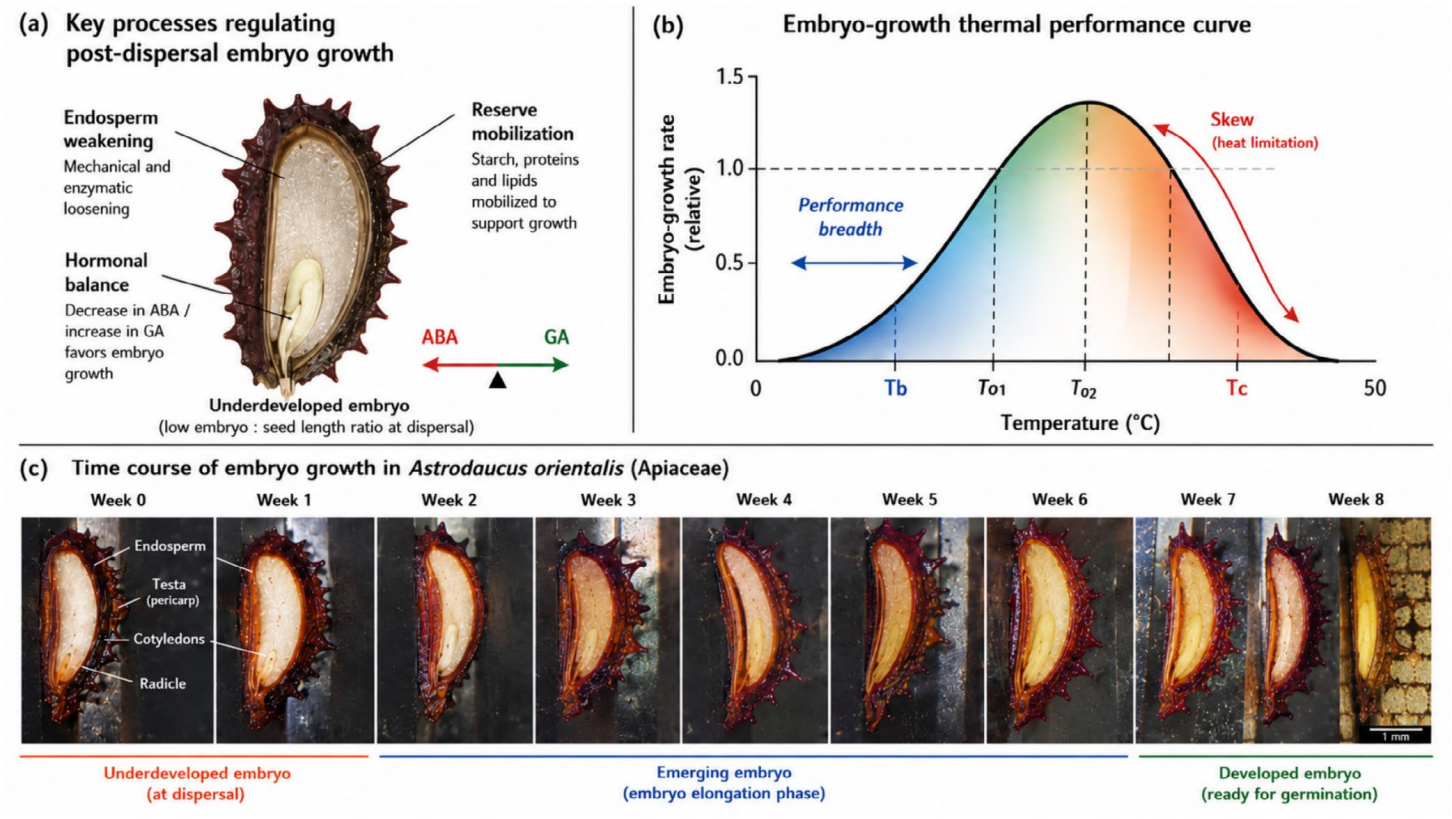
Embryo growth is governed by a temperature‐dependent performance curve that is distinct from germination. (a) Post‐dispersal embryo growth in species with underdeveloped embryos is regulated by a suite of coordinated, temperature‐sensitive processes, including weakening of surrounding tissues (endosperm, testa, or pericarp), mobilization of stored reserves, and shifts in hormonal balance between abscisic acid (ABA) and gibberellins (GA). Following imbibition, embryo growth is often preceded by a latent phase, after which elongation proceeds at temperature‐dependent rates. (b) This developmental phase can be formalized as a thermal performance curve (TPC) characterized by a base temperature (*T*_b_) below which growth is negligible, an optimal temperature range (*T*_o1_–*T*_o2_) over which elongation rates are maximized, and an asymmetric decline at supra‐optimal temperatures (*T*_c_), reflecting performance breadth and skew. (c) Representative time‐series image illustrates post‐dispersal embryo elongation (*Astrodaucus orientalis* (Apiaceae)—unpublished data; photograph by Elias Soltani).

Thermal performance curves (TPCs) provide a powerful framework for understanding how biological rates respond to temperature, capturing key parameters such as base (*T*_b_), optimum (*T*_o_), and ceiling (*T*_c_) temperatures, as well as performance breadth and skew (Padfield et al. 2021; Wooliver et al. 2022). Although TPCs are routinely applied to germination, seedling growth, and physiological processes (Bewley et al. 2013; Maleki, Soltani, et al. 2024), they have rarely been used to conceptualize embryo growth itself. This omission is increasingly untenable, because it implicitly treats embryo growth as a timing artifact or a passive extension of dormancy release, rather than as a temperature‐dependent developmental process with its own constraints, thresholds, and failure modes. Empirical evidence now indicates that embryo elongation exhibits well‐defined thermal responses that are not trivially predicted by germination behavior (Blandino et al. 2025; Walker et al. 2026). As illustrated in Figure 1, embryo growth in taxa with underdeveloped embryos depends on the coordinated progression of multiple processes, including weakening of surrounding tissues, mobilization of endosperm reserves, and shifts in hormonal balance, particularly the antagonistic regulation by abscisic acid (ABA) and gibberellins (GA) (Bewley et al. 2013; Steinbrecher and Leubner‐Metzger 2017). Each of these components is temperature sensitive, such that embryo elongation emerges from their joint thermal regulation rather than from temperature acting on growth rate alone.

Critically, the timing of embryo growth relative to imbibition varies among species and dormancy classes. In some species, particularly those exhibiting MPD, embryo growth may be preceded by a latent phase whose duration depends on prior thermal history, dormancy status, and hormonal context. In others, embryo elongation may begin shortly after imbibition once suitable environmental conditions are encountered (Baskin and Baskin 2014). Regardless of the specific sequence of events, embryo growth ultimately proceeds at temperature‐dependent rates and may therefore be viewed as a thermal performance process rather than a simple time‐dependent delay (Steinbrecher and Leubner‐Metzger 2017). This temporal structure is consistent with a TPC‐governed developmental process, in which both the onset and rate of embryo growth are constrained by temperature, rather than with a simple time‐dependent delay (Donohue et al. 2015; Wooliver et al. 2022). Framing embryo growth within a TPC framework therefore reveals it as a discrete thermal checkpoint that actively filters regeneration, shaping when, and under which climatic conditions, seeds can complete development and successfully transition to germination phase (Vandelook et al. 2009).

## 3 The Embryo Growth Thermal Niche (EGTN)

**FIGURE 2:**
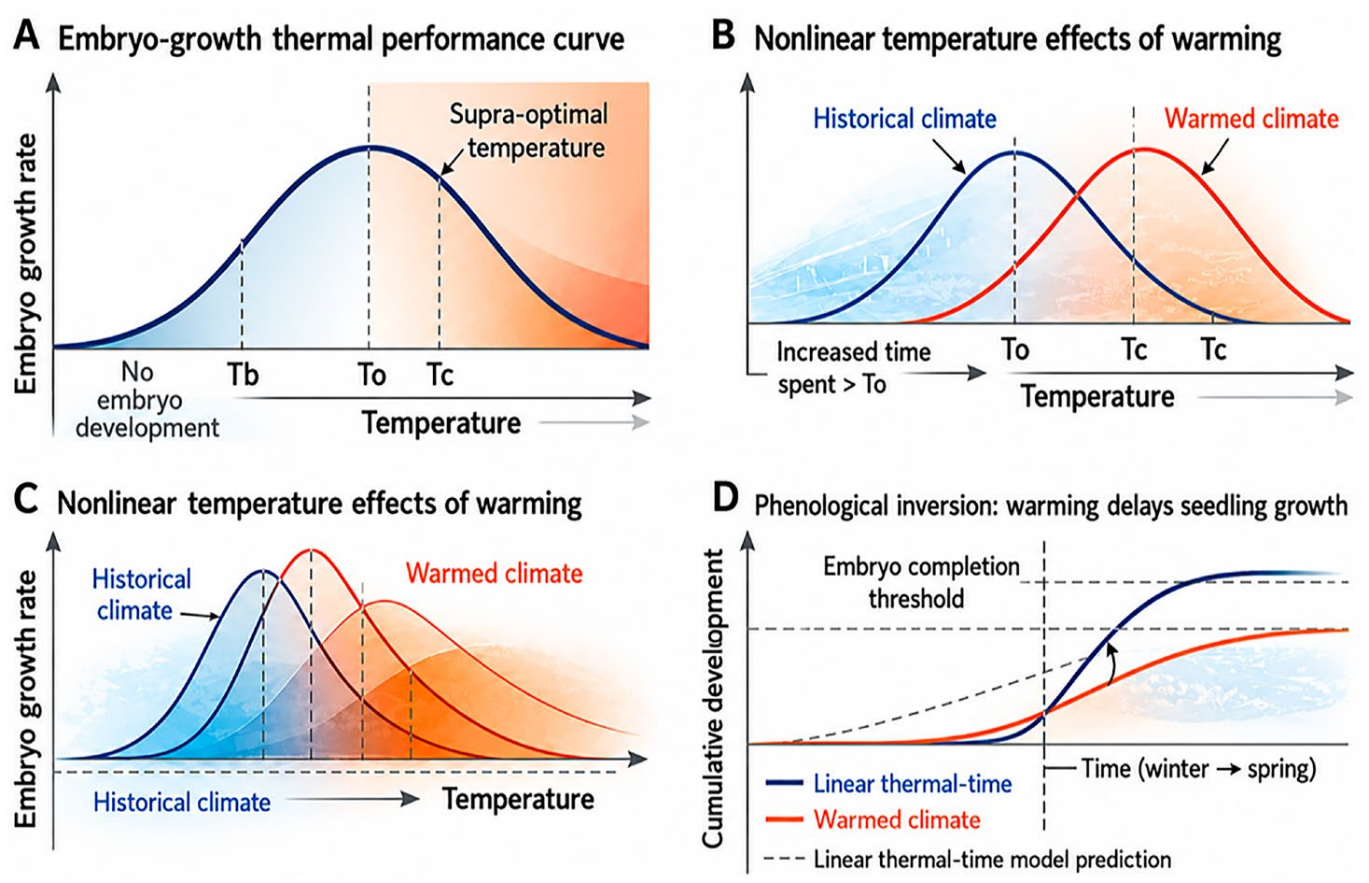
It is assumed that embryo growth follows a nonlinear thermal performance curve defined by a base temperature (*T*_b_), optimum (*T*_o_), and an upper thermal limit (*T*_c_). Growth rates increase up to *T*_o_ but decline sharply at supra‐optimal temperatures, such that warming beyond *T*_o_ does not accelerate development. (B) Under historical climates, temperature distributions overlap primarily with the accelerating portion of the embryo‐growth thermal performance curve. (C) Climate warming shifts temperature distributions toward higher means and increased variance, increasing the proportion of time seeds experience supra‐optimal temperatures where embryo growth is inefficient or arrested. (D) As a consequence, cumulative embryo growth proceeds more slowly under warmer conditions, delaying attainment of the embryo completion threshold and shifting seedling emergence later rather than earlier, a phenological inversion relative to predictions from linear thermal‐time models (dashed line).

We advance a thermal performance–based framework that conceptualizes post‐dispersal embryo growth as a discrete regeneration trait governed by two coupled temperature filters. In species with MPD, embryo growth may be preceded by physiological dormancy release, which can require distinct environmental cues and may occur before, during, or concurrently with embryo development (Baskin and Baskin 2014). Our framework focuses specifically on the embryo‐growth phase itself. Once embryo growth becomes developmentally possible, it occurs within a species‐specific thermal window bounded by a lower thermal limit (*T*_b_), an optimum temperature range (*T*_o_), and an upper thermal limit (*T*_c_), reflecting the coordinated hormonal and mechanical processes that permit embryo elongation (Finch‐Savage and Leubner‐Metzger 2006; Wang et al. 2025). Growth then proceeds according to a thermal performance curve in which temperature determines both growth rate and thermal tolerance (Figure 1a; Walker et al. 2026). Together, these components define the embryo growth thermal niche (EGTN), which is conceptually distinct from germination thermal responses (see Bradford and Bello 2022 for detailed discussion on germination thermal modeling). Under this framework, embryo growth can be developmentally active yet ecologically ineffective if initiation is delayed or if growth occurs near or beyond thermal ceilings (Wawrzyniak et al. 2023). In this sense, the EGTN defines not only the pace of embryo growth but also the climatic window within which regeneration is possible (Figure 1a–c). Although the relative importance of embryo‐growth thermal requirements will vary among species and environments, these requirements may constrain regeneration timing by limiting the environmental conditions under which embryo development can be completed. Critically, the EGTN predicts regeneration responses to warming that diverge fundamentally from rate‐only thermal time models, including delayed or inverted phenological outcomes under increasingly warm winters (Figure 2).

Although temperature represents the primary axis of the embryo‐growth thermal niche, other environmental cues may modify embryo‐growth responses. In particular, light can influence the initiation, progression, or completion of embryo growth in some species with underdeveloped embryos. For example, light has been shown to regulate embryo growth and subsequent germination in species with MD and MPD, often interacting with temperature and dormancy status (Baskin and Baskin 1988; Vandelook et al. 2007). More recently, Di Stefano et al. (2025) demonstrated that embryo growth in Mediterranean temporary‐pond species was strongly promoted by light and substantially reduced under darkness. These observations suggest that the EGTN is embedded within a broader multidimensional regeneration niche in which temperature defines the thermal boundaries for embryo development, while light may act as a positional or habitat‐quality cue that modifies the probability or rate of embryo elongation. Future applications of the EGTN framework should therefore consider environmental interactions, particularly temperature × light effects, when sufficient empirical data are available.

## 4 Embryo‐Growth Strategies as Climate Filters

Viewing embryo growth through a thermal performance lens reveals its role in structuring regeneration strategies, rather than merely regulating growth rate. Species differ not only in whether embryos must grow post‐dispersal, but in how embryo‐growth TPCs filter climatic risk (Figure 2). We hypothesize that species with low optimum temperatures and narrow thermal performance breadths may exhibit relatively conservative embryo‐growth strategies, restricting development to a narrower range of thermal conditions. Conversely, species with broader embryo‐growth TPCs and higher thermal ceilings may permit embryo growth across a wider range of temperatures. The ecological consequences of these strategies, however, are likely to depend on the environmental context in which they operate. Because temperature is often coupled with other factors such as moisture availability, seasonality, flooding, or disturbance, the relationship between embryo‐growth thermal traits and regeneration opportunities may vary substantially among biomes and climatic regimes (Vandelook et al. 2009, 2024). By contrast, species with broader embryo‐growth TPCs and higher thermal ceilings exhibit thermally permissive strategies, allowing embryo growth across a wider range of thermal environments (Wooliver et al. 2022; see also Figure 2).

These embryo‐growth strategies are analogous to, but mechanistically distinct from, classical germination strategies such as bet‐hedging or best‐bet, operating upstream of radicle emergence and therefore reshaping regeneration outcomes even when dormancy and germination cues are satisfied (Pausas et al. 2022). Importantly, embryo‐growth thermal niches are expected to vary among species within the same dormancy class. Thus, although dormancy class defines the broader developmental context in which embryo growth occurs, species sharing similar dormancy types or dormancy‐release requirements may nevertheless differ substantially in regeneration timing if their embryo‐growth thermal niches differ. In this sense, the EGTN framework is intended to complement, rather than replace, existing dormancy classifications by explicitly considering the thermal ecology of embryo development (Maleki, Soltani, et al. 2024; Figure 1b). A critical and underappreciated constraint emerges at the upper end of the thermal spectrum. In many systems, the *T*_c_ for growth occurs at temperatures already common during late winter or early spring (Sentinella et al. 2020; Maleki, Soltani, et al. 2024; Maleki, Chmielarz, et al. 2024). Under such conditions, warming does not accelerate regeneration but instead slows or arrests embryo growth as temperatures approach or exceed *T*_c_, a process already documented for germination (Sentinella et al. 2020). Embryo‐growth TPCs therefore act as thermal risk filters, shaping regeneration strategies in ways not captured by dormancy or germination traits alone.

## 5 Climate Change and Phenological Mismatch

The EGTN framework generates the hypothesis that climate warming may, under some conditions, delay regeneration even when germination rates increase. Warming is often assumed to accelerate development through faster thermal‐unit accumulation, but this expectation holds primarily when temperatures remain within the accelerating portion of a species' thermal response curve (Pawar and Kontopoulos 2025). We propose that in species with underdeveloped embryos, warming could reduce cumulative embryo development if temperatures increasingly occur near or beyond thermal optima or upper thermal limits. Under such circumstances, additional warming may not translate into faster embryo growth despite increased thermal exposure (Wooliver et al. 2022). The magnitude and direction of this response are expected to depend on species‐specific thermal performance curves and the climatic context in which they operate.

Figure 2 illustrates the mechanistic basis of this prediction. Embryo growth is temperature‐dependent and typically follows a unimodal thermal response, although the exact functional form may vary among species, ranging from smooth nonlinear curves to segmented or dent‐like patterns, as observed in germination and other plant processes. In many cases, growth rates increase toward an optimum and then decline sharply at supra‐optimal temperatures, particularly when Tc is relatively low (Figure 2a; see also Angilletta Jr 2006). In systems where seasonal temperatures historically overlap with the accelerating portion of the embryo‐growth thermal response, developmental progress may accumulate steadily through time (Figure 2b). If warming shifts temperature distributions toward values approaching or exceeding thermal optima or upper thermal limits, embryo growth could slow despite higher average temperatures (Figure 2c). Under these conditions, warming may delay rather than advance completion of embryo development, potentially producing phenological outcomes that differ from those predicted by linear thermal‐time models (Figure 2d). Such responses are expected to be particularly relevant in temperate and Mediterranean systems where embryo growth often occurs during cool seasons and where warming may increase the frequency of temperatures near upper thermal limits for development (Sentinella et al. 2020; Maleki, Chmielarz, et al. 2024). Whether similar patterns occur across other biomes and climatic regimes remains largely unknown and represents an important avenue for future research.

Embryo‐growth thermal performance curves often exhibit strong curvature and relatively low upper thermal limits, such that small increases in temperature or variance can generate disproportionately large reductions in embryo growth rate (Donohue et al. 2015; Blandino et al. 2025; Walker et al. 2026). Because the thermal response of embryo growth remains insufficiently characterized, it may follow linear, segmented, or nonlinear forms depending on species and temperature range. Consequently, reliance solely on linear thermal‐time models or germination‐based predictions may not fully capture embryo‐growth dynamics, particularly if the underlying response deviates from linearity. Both linear and nonlinear frameworks may therefore be informative, but their applicability should be evaluated empirically. Such model misspecification could help explain why observed phenological shifts sometimes deviate from expectations based only on germination responses (Bradford and Bello 2022). At present, empirical data describing embryo‐growth thermal performance curves remain limited and concentrated in a relatively small number of taxa and climatic regions. Consequently, the prevalence of phenological inversion and other warming‐induced responses remains uncertain. We therefore view these predictions as testable hypotheses rather than general outcomes, and broader comparative studies across biomes will be necessary to evaluate their generality.

## 6 Evolutionary Constraint and Phylogenetic Signal

If embryo‐growth TPCs emerge from tightly integrated interactions among hormonal regulation, tissue mechanics, and developmental architecture (Finch‐Savage and Leubner‐Metzger 2006), they are expected to be subject to strong evolutionary constraint. Unlike germination responses, which can evolve relatively rapidly through shifts in dormancy depth or sensitivity to environmental cues (Willis et al. 2014), embryo‐growth TPCs are embedded within conserved aspects of seed structure and developmental timing, potentially limiting their evolutionary lability (Carta et al. 2024). Emerging comparative evidence supports this view, indicating a measurable phylogenetic footprint in embryo size, growth requirements, and temperature sensitivity across angiosperms (Visscher et al. 2022; Vandelook and Carta 2025). Crucially, this phylogenetic signal cannot be reduced to general climatic niche conservatism (Losos 2008). Climatic niche conservatism typically reflects broad‐scale correlations between species distributions and macroclimatic conditions (Qiao et al. 2024; Riera et al. 2025), whereas the EGTN operates at a finer developmental scale, constraining regeneration even when climatic conditions fall within the species' realized niche and germination cues are satisfied (Vandelook et al. 2024). Thus, two species occupying similar thermal environments, and even exhibiting comparable germination temperature optima, may differ markedly in regeneration timing if their embryo‐growth TPCs diverge (Figures 1 and 2).

A concrete comparative prediction follows from this distinction: lineages in which underdeveloped embryos evolved early and have been retained over deep evolutionary time, such as Ranunculales or Apiaceae (Forbis et al. 2002; Baskin and Baskin 2023), are expected to exhibit stronger conservatism in embryo‐growth TPC parameters (particularly *T*_c_ and performance breadth) than lineages in which embryo reduction arose more recently or has been repeatedly lost (Vandelook and Carta 2025). In these clades, shifts in flowering phenology, dispersal timing, or dormancy‐breaking cues may occur without corresponding shifts in embryo‐growth thermal limits, generating persistent regeneration bottlenecks under changing climates. By contrast, lineages characterized by fully developed embryos at dispersal are predicted to lack this constraint, as regeneration proceeds directly from germination without an intervening embryo‐growth checkpoint (Baskin and Baskin 2014). Such evolutionary response has profound implications under rapid climate warming. If embryo‐growth performance breadth evolves more slowly than ambient temperatures, warming may increasingly expose seeds to supra‐optimal conditions during the embryo‐growth phase, even in regions where adult plants remain climatically well matched (Figure 2). This decoupling increases the likelihood of regeneration failure or phenological mismatch driven not by distributional limits, but by inherited developmental thresholds (Donohue et al. 2015). In this sense, embryo‐growth thermal niches represent a hidden axis of evolutionary vulnerability, one that complements, but is mechanistically distinct from, classical climatic niche conservatism.

## 7 Predictions and Future Directions

The EGTN framework generates a forward‐looking research agenda that repositions post‐dispersal embryo growth as a central determinant of regeneration in seeds with underdeveloped embryo (MD and MPD) under global change. First, embryo‐growth thermal performance curves are predicted to be only weakly correlated with germination TPCs across species, reflecting their distinct physiological controls and selective histories (Figure 1). Comparative analyses that collapse embryo growth into germination are therefore expected to underestimate regeneration diversity and systematically misclassify climate sensitivity. Second, species characterized by narrow embryo‐growth performance breadths or low *T*_c_ should be disproportionately vulnerable to warming, particularly in systems where regeneration occurs during winter or early spring (Figure 2). In such taxa, warming is predicted to delay regeneration or reduce recruitment even as germination rates seem to increase, generating outcomes inconsistent with linear thermal‐time expectations. Third, experimental manipulation of hormonal pathways (e.g., GA_3_ application or altered ABA sensitivity) is expected to shift the position or shape of embryo‐growth TPCs but rarely eliminate upper thermal constraints, indicating that ceiling temperatures emerge from integrated developmental architecture rather than simple hormonal limitation. Finally, embryo‐growth thermal niches are expected to exhibit a strong phylogenetic footprint, potentially constraining adaptive responses under rapid climate change. Nevertheless, species with broad geographic distributions may exhibit substantial intraspecific variation and local adaptation in embryo‐growth thermal parameters, allowing populations to respond differently to regional climatic conditions. Understanding the balance between phylogenetic conservatism and population‐level adaptation therefore represents an important avenue for future research. Lineages characterized by thermally conservative embryo strategies may therefore face systematic regeneration mismatches as climates warm, representing a previously unrecognized axis of evolutionary vulnerability. Together, these predictions establish embryo growth not as a secondary modifier of germination, but as a primary determinant of regeneration timing, climate sensitivity, and evolutionary constraint.

## 8 Concluding Remarks

By reframing post‐dispersal embryo growth as a thermal performance trait defining a regeneration niche, the EGTN framework integrates developmental biology, germination strategy and climate sensitivity into a unified perspective. This shift moves embryo growth from the periphery of seed biology to a central position in predicting plant regeneration under global change. Quantifying embryo‐growth thermal niches across taxa with underdeveloped embryo at dispersal will be essential for forecasting regeneration success, identifying evolutionary constraints and anticipating phenological responses in a warming world.

## Author Contributions

**Keyvan Maleki:** conceptualization (equal), writing – original draft (equal), writing – review and editing (equal). **Elias Soltani:** conceptualization (equal), writing – original draft (equal), writing – review and editing (equal).

## Funding

The authors have nothing to report.

## Conflicts of Interest

The authors declare no conflicts of interest.

## Data Availability

This article is a viewpoint and does not report original experimental data or generate new datasets. All information discussed is derived from previously published literature, which has been appropriately cited within the manuscript. Therefore, no additional data are available.
